# Embryonic oxygen enhances learning ability in hatchling lizards

**DOI:** 10.1186/1742-9994-11-21

**Published:** 2014-03-03

**Authors:** Bao-Jun Sun, Ting-Ting Wang, David A Pike, Liang Liang, Wei-Guo Du

**Affiliations:** 1 Key Laboratory of Animal Ecology and Conservational Biology, Institute of Zoology, Chinese Academy of Sciences, Beijing 100101, People’s Republic of China; 2 School of Life Science, Anhui University, Hefei 230039, People’s Republic of China; 3 Centre for Tropical Ecological and Sustainability Science, School of Marine and Tropical Biology, James Cook University, Townsville, QLD 4811, Australia

**Keywords:** Embryonic development, Oxygen concentration, Cognitive ability, Mongolian Racerunner lizard, *Eremias argus*

## Abstract

**Introduction:**

Producing smart offspring is an important fitness trait; individuals with enhanced cognitive ability should be more adept at responding to complex environmental demands. Cognitive ability can be influenced by conditions experienced during embryonic development. Although oxygen is necessary for embryonic development, availability can be limited within the nest environment because of substrate type, hydric conditions, and temperature. We do not yet understand, however, whether oxygen availability during embryonic development influences offspring fitness, especially cognitive ability. To address this question we incubated Mongolian Racerunner lizard (*Eremias argus*) eggs under hypoxic (12% O_2_), normoxic (21% O_2_), and hyperoxic conditions (30% O_2_).

**Results:**

Hypoxia not only slowed hatching time, but also resulted in constrained cognitive ability relative to hatchlings experiencing normoxic or hyperoxic incubation conditions. Oxygen did not influence hatching success, body size or sprint speed of hatchlings.

**Conclusions:**

Oxygen availability during embryonic development has important influences on incubation duration and cognitive ability of hatchling lizards. This study provides the first evidence that oxygen availability during embryonic development can modify cognitive ability of oviparous reptiles.

## Introduction

During embryonic development the phenotype is integrated through a combination of genetic, maternal and environmental effects (reviewed by [1,2]). The incubation environment of oviparous species, in which the embryos develop outside of the mother’s body, can vary substantially depending on the nest environment selected and whether or not parental care is provided [1-3]. Attributes such as the thermal and hydric conditions during incubation can have profound effects on hatchling morphology, locomotion, growth and survival [3-5]. Despite a broad understanding of how attributes of the nest environment influence offspring morphology and performance (e.g., sprint speed), we know much less about whether the primary drivers of embryonic development influence cognition and behaviour, and how they work.

Cognitive ability is a flexible behavioural trait that can be modified by the incubation environment [6,7]. Naïve animals, such as those emerging from eggs, must be able to learn about the complex environment efficiently; for example, the ability to locate safe retreat sites to protect against potential predators or unfavourable microenvironments [8]. Individuals with enhanced cognitive ability may be able to respond more adaptively to complex, and sometimes novel, environmental demands [7,9], which may result in strong selective pressure for individuals that are able to quickly evaluate and respond to ecological challenges.

Oxygen is an essential environmental factor required for embryonic development, which oviparous species obtain from the nest environment by diffusion through the eggshell [10-12]. Oxygen availability within natural nests can vary significantly due to metabolic activities of microbes and consumption of oxygen by other incubating eggs [13,14]. For example, in green sea turtle nests, oxygen partial pressure declines significantly towards the end of incubation, as developing embryos grow larger [15]. The oxygen concentration experienced during embryonic development can have profound impacts on hatchling morphology, growth and metabolic rates, as has been demonstrated in alligators (*Alligator mississippiensis*) [12]. How oxygen influences other fitness determinants, such as cognition, is still unknown.

We incubated Mongolian Racerunner lizard (*Eremias argus*) eggs under different oxygen concentrations, and examined the consequences on hatchling phenotype and cognitive ability. Mongolian Racerunners have small home ranges in nature, and when confronted with potential threats individuals escape into safe retreat sites [16]. Spatial cognition of safe retreat sites (from predators, unfavourable conditions, etc.) is thus likely to be an important fitness trait [8]. By manipulating oxygen availability during egg incubation, we aim to elucidate the direct consequences on the cognitive ability of hatchling lizards.

## Results

Initial egg mass did not differ significantly among oxygen treatments (Table 1). Incubation duration was significantly shorter under hyperoxic conditions, but oxygen did not significantly influence hatching success, body size, or survival (Table 1).

**Table 1:** **Statistical comparisons among hatchling *Eremias argus *** **lizards incubated under hypoxic (12% O**_**2**_**), normoxic (21% O**_**2**_**), or hyperoxic (30% O**_**2**_**) conditions**

**Attribute**	**Oxygen concentration during incubation**			**Test statistic**	***P*-value**
	**Hypoxic**	**Normoxic**	**Hyperoxic**		
	**(12% O**_**2**_**)**	**(21% O**_**2**_**)**	**(30% O**_**2**_**)**		
Initial egg mass (g)	0.466 ± 0.012	0.440 ± 0.013	0.459 ± 0.014	*F*_2,51_ = 1.093	0.343
Incubation duration (days ± SE)	35.67 ± 0.19^a^	35.17 ± 0.19^a^	34.86 ± 0.23^b^	*F*_2,44_ = 3.896	**0.028**
Egg hatching success (%, n)	88.24 (15/17)	90.00 (18/20)	77.88 (14/18)	χ^2^ = 1.292	0.524
Initial SVL (mm ± SE)	28.21 ± 0.325	27.72 ± 0.320	27.73 ± 0.605	*F*_2,44_ = 0.440	0.647
30-day SVL (mm ± SE)	33.68 ± 0.588	32.44 ± 0.758	33.25 ± 0.698	*F*_2,31_ = 0.822	0.449
Initial mass (g ± SE)	0.56 ± 0.015	0.54 ± 0.016	0.59 ± 0.014	*F*_2,43_ = 1.653	0.203
30-day mass (g ± SE)	0.93 ± 0.024	0.82 ± 0.058	0.96 ± 0.046	*F*_2,30_ = 1.483	0.243
Survival to 30 days (%, n)	66.67 (10/15)	72.22 (13/18)	78.57 (11/14)	χ^2^ = 0.513	0.774

^a,b^means with different superscripts are statistically different (Tukey's test).

Statistically significant results are shown in bold.

**Figure 1:**
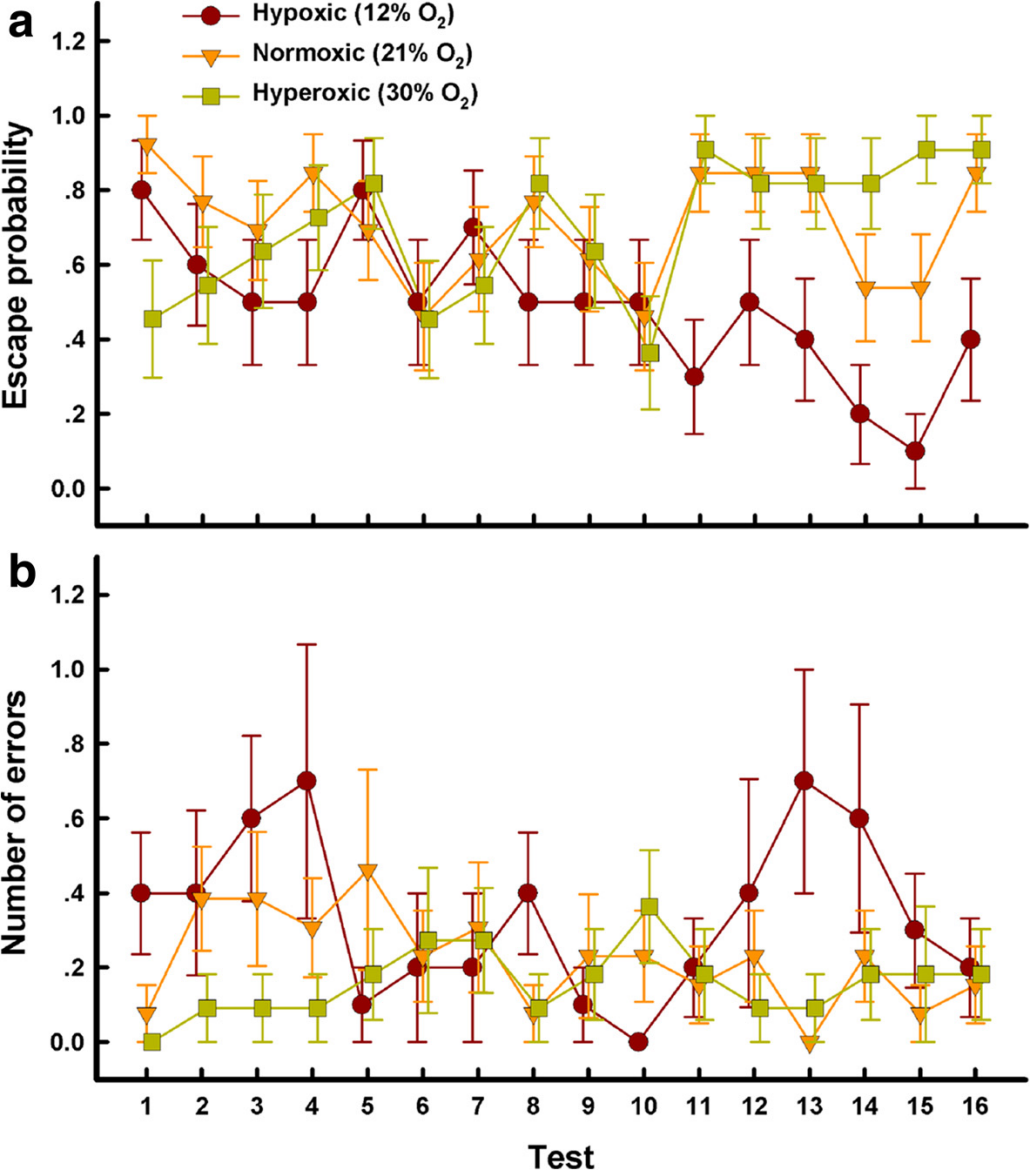
**The ability of hatchling *Eremias argus *lizards to improve their location of safe retreat sites over time, expressed as escape probability (a), and the number of errors made in selecting shelter sites (b).** Hatchlings were incubated under hypoxic (12% O_2_), normoxic (21% O_2_), or hyperoxic (30% O_2_) conditions. Data are presented as the mean ± SE, and the sample sizes are 10, 13 and 11 for hypoxic, normoxic and hyperoxic treatment groups, respectively.

Both oxygen concentration and timing of testing significantly influenced learning processes (Table 2; with an Independent working correlation matrix, Table 3). The escape probability of lizards incubated under hyperoxic condition was significantly higher than hypoxic condition, but was not different from normoxic incubation treatment (Figure 1a; Table 4). As our cognitive testing progressed, the escape probabilities decreased in those lizards that were incubated under hypoxic conditions (B = -0.126, R^2^ = 0.101 *χ*^2^ = 11.668, *df* = 1, *p* = 0.001), but were enhanced in lizards under hyperoxic environments (B = 0.093, R^2^ = 0.052 *χ*^2^ = 6.312, *df* = 1, *p* = 0.012), and did not change in lizards under the normoxic conditions (*p* = 0.457) (Figure 1a). The number of errors that hatchling lizards made was significantly influenced by both oxygen concentration and timing of testing (Table 2; with Independent working correlation matrix, Table 3). Lizards incubated under hyperoxic conditions made fewer errors than those incubated under hypoxic conditions, but did not differ from the normoxic conditions (Figure 1b; Table 4). The error rates did not change during learning processes in any of the treatments (low oxygen, *p* = 0.738; normal oxygen, *p* = 0.381; high oxygen, *p* = 0.093) (Figure 1b). We found no significant differences between the sexes in their escape probability or error rates (Table 2).

**Table 2:** Results of generalized estimating equations (GEE) in testing the effects of oxygen concentration and sex on lizard escape outcome and the number of errors made throughout the experimental period

	**Wald *χ***^**2**^	***df***	***P***
**Escape**			
Intercept	36.121	1	**<0.001**
Oxygen concentration	13.647	2	**0.001**
Sex	0.337	1	0.562
Test timing	31.342	15	**0.008**
**Number of errors**			
Intercept	22.541	1	**<0.001**
Oxygen concentration	11.116	2	**0.004**
Sex	0.456	1	0.499
Test timing	46.413	15	**<0.001**

Statistically significant results are shown in bold.

**Table 3:** QIC and QICC value of each potential working correlation matrix

	**QIC**	**QICC**
**Escape**		
Independent	710.544	**703.414**
AR 1	710.649	703.429
Exchangeable	710.638	703.442
M-Dependent	710.625	703.428
Unstructured	711.16	708.265
**Number of errors**		
Independent	57.859	**47.347**
AR 1	56.133	47.392
Exchangeable	57.518	47.364
M-dependent	57.103	47.393
Unstructured	39.784	48.057

The lowest QICC among models are shown in bold. The ‘Independent working correlation matrix’ was selected when running our analysis.

**Table 4:** Parameter estimates, confidence intervals and hypothesis tests of results in generalized estimating equations (GEE)

	**Estimate**	**SE**	**95% CI**		**Hypothesis test**		
			**Lower**	**Upper**	**Wald χ**^**2**^	***df***	***P***
**Escape**							
Intercept	1.247	0.530	0.209	2.285	5.543	1	**0.019**
Oxygen concentrations							
Hypoxic (12% O_2_)	-0.960	0.340	-1.625	-0.295	7.995	1	**0.005**
Normoxic (21% O_2_)	0.053	0.377	-0.686	0.793	0.020	1	0.888
Hyperoxic (30% O_2_)	0^a^						
Sex	-0.165	0.284	-0.721	0.392	0.337	1	0.562
Test timing							
Test-1	0.153	0.506	-0.839	1.145	0.091	1	0.763
Test-2	-0.282	0.566	-1.391	0.827	0.249	1	0.618
Test-3	-0.415	0.459	-1.315	0.485	0.818	1	0.366
Test-4	0.000	0.555	-1.087	1.087	0.000	1	1.000
Test-5	0.317	0.591	-0.842	1.475	0.287	1	0.592
Test-6	-0.919	0.465	-1.829	-0.008	3.910	1	0.048
Test-7	-0.415	0.499	-1.393	0.563	0.693	1	0.405
Test-8	0.000	0.555	-1.087	1.087	0.000	1	1.000
Test-9	-0.415	0.456	-1.309	0.478	0.829	1	0.362
Test-10	-1.166	0.562	-2.267	-0.065	4.309	1	**0.038**
Test-11	0.000	0.419	-0.822	0.822	0.000	1	1.000
Test-12	0.153	0.457	-0.742	1.048	0.112	1	0.738
Test-13	0.000	0.514	-1.007	1.007	0.000	1	1.000
Test-14	-0.795	0.408	-1.596	0.005	3.789	1	0.052
Test-15	-0.919	0.469	-1.838	0.001	3.833	1	**0.050**
Test-16	0^a^						
**Number of errors**							
Intercept	-0.094	0.051	-0.195	0.007	3.336	1	0.068
Oxygen concentrations							
Hypoxic (12% O_2_)	0.249	0.075	0.102	0.396	10.980	1	**0.001**
Normoxic (21% O_2_)	0.050	0.060	-0.068	0.168	0.687	1	0.407
Hyperoxic (30% O_2_)	0^a^						
Sex	-0.052	0.076	-0.201	0.098	0.456	1	0.499
Test timing							
Test-1	-0.090	0.080	-0.246	0.067	1.257	1	0.262
Test-2	0.097	0.084	-0.069	0.262	1.318	1	0.251
Test-3	0.155	0.121	-0.082	0.391	1.648	1	0.199
Test-4	0.374	0.138	0.104	0.645	7.379	1	**0.007**
Test-5	0.475	0.233	0.017	0.932	4.141	1	**0.042**
Test-6	0.326	0.143	0.046	0.605	5.224	1	**0.022**
Test-7	0.291	0.126	0.044	0.537	5.339	1	**0.021**
Test-8	-0.056	0.083	-0.218	0.106	0.457	1	0.499
Test-9	0.229	0.172	-0.107	0.566	1.781	1	0.182
Test-10	0.102	0.055	-0.005	0.210	3.494	1	0.062
Test-11	0.017	0.055	-0.091	0.124	0.091	1	0.762
Test-12	0.269	0.184	-0.091	0.629	2.141	1	0.143
Test-13	0.174	0.208	-0.234	0.581	0.699	1	0.403
Test-14	0.169	0.145	-0.116	0.453	1.351	1	0.245
Test-15	0.158	0.204	-0.241	0.558	0.602	1	0.438
Test-16	0^a^						

^a^parameter estimates are redundant; CI, confidence interval.

Statistically significant results are shown in bold.

## Discussion

Using established methods in our spatial cognitive tests [7], lizards should demonstrate learning by two metrics: both increased escape probability and decreased errors over 16 consecutive tests. Oxygen availability during incubation significantly influenced escape probability, but did not significantly influence the errors rates of hatchling Racerunner lizards over the tests (Figure 1; Table 4). Locating safe retreat sites is an important fitness trait, because individuals escape into safe retreat sites when confronted with potential threats [8,16]. Despite the lizards incubated under hyperoxic conditions making fewer errors than lizards under hypoxic environments, the spatial learning results are equivocal because errors rates did not vary temporally within any oxygen treatment. Therefore, this may reflect a learning outcome rather than spatial learning process. Mammals and birds can strategically locate specific goals, evidence of spatial learning [17]. In the squamates, however, some species navigate using beacons, whereas others use trial-and-error searching [18,19]. For hatchling lizards, goal-oriented tasks may be most relevant to immediate fitness (i.e. locating safe retreat sites, as tested in this study) and thus understanding whether these patterns are due to associative or spatial learning is not as important [20]. Although we were unable to confirm the learning strategy used by lizards, we demonstrate an increase in escape probability over consecutive tests in lizards incubated under hyperoxic conditions. Oxygen is widely known to influence the cognitive abilities of mammals [21,22], and our study provides the first evidence that oxygen can modify cognitive ability, and thus fitness, in oviparous reptiles.

Hatchling lizards incubated under hypoxic environments did not increase their escape probability; instead, these lizards decreased their probability of successfully locating the open retreat and constantly made high error rates across tests (Figure 1a,b). Nevertheless, the temperature-dependent physiology of Mongolian Racerunners [23] may allow some hatchlings to compensate for reduced cognition by maintaining higher body temperatures, potentially overcoming an intrinsically weak ability to learn the location of safe retreats by increasing escape speed when confronted with potential threats [7].

The mechanism of how oxygen concentration influences cognition is unclear, although brain size and brain structure may be responsible for these differences [24,25]. For example, avian hippocampus size is correlated with spatial learning ability [26] and experience [27]. By comparison, reptiles do not have hippocampus, however the medial cortex is assumed as a homologue [17]. Larger brains may be better able to deal with environmental challenges, such as successfully colonizing novel habitats after undergoing human translocation [9]. Oxygen availability can also influence hormone levels during brain development, potentially modifying memory retention or learning ability [28,29]. Embryonic development of oviparous reptiles is positively related to metabolic rates, suggesting that aerobic metabolism could be another cause [12,30]. Hypoxia during incubation may impose restrictions on brain development through embryonic metabolic pneumatorexis [e.g., [31], thereby limiting brain size development or brain structure formation.

Enhanced cognitive ability may increase survival rates and reproductive success by facilitating adaptive responses in complex environments [7]. Because hatchlings are extremely vulnerable to predation [32], natural selection should favour rapid learning of safe retreat sites very early in life. Individuals that take longer to select safe retreats or find suboptimal sites may suffer increased predation. Further work integrating multiple aspects of the incubation environment (e.g., oxygen and temperature/moisture) will provide greater insight into the main factors driving the organismal phenotype, including cognition.

## Conclusions

We investigated the effects of oxygen availability during embryonic development on the cognitive ability of hatchling lizards and showed that increased oxygen levels during incubation enhanced cognition. Our study provides the first evidence that oxygen can modify cognitive ability in oviparous reptiles, and highlights the neglected importance that developmental oxygen can have on animals after hatching from the egg.

## Materials and methods

We collected gravid female *E. argus* from Jungar Banner, Inner Mongolia (40°12’17”N, 111°07’43”E; 1036 m elevation) during June 2013 and maintained them individually in terraria (310 × 210 × 180 mm) lined with moist vermiculite (20 mm thick, water potential = -220 kpa) under a 14L:10D photoperiod at 24 ± 1°C. Supplemental heating was available from 0800 to 1600 hours. Food (crickets dusted with vitamin supplements) and water were available *ad libitum*.

We checked terraria 4 times daily for freshly laid eggs. Eggs were collected between 14-22 June and the collected eggs were weighed (±0.001 g) immediately to minimize initial mass changes due to water exchange. Although the clutch size of female *E. argus* ranges from two to five [16], we only used one egg from each clutch in this study to eliminate genetic effects. We randomly allocated a single egg from 55 females into hyperoxic (30% O_2_: n = 18 eggs), normoxic (21% O_2_: n = 20), or hypoxic (12% O_2_: n = 17) incubation conditions. Eggs were incubated at 28 ± 0.5°C with 6-8 eggs placed inside a vermiculite-filled container (-220kpa) which was sealed inside a 40L vacuum bag filled with compressed oxygen and nitrogen [12]. Air was renewed every second day to maintain incubation conditions ± 2% of desired.

**Figure 2:**
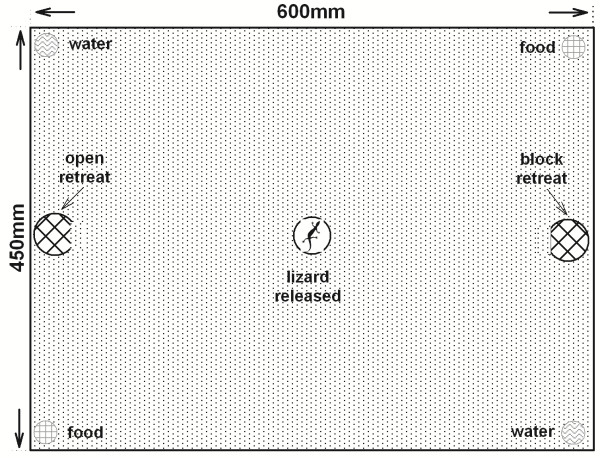
**The terrarium setup used in husbandry of hatchling lizards and in tests of cognitive ability.** Food and water were available *ad libitum* during husbandry, but these resources and all conspecifics were removed from the terraria during tests of cognitive ability.

Towards the end of incubation, we checked the containers twice daily (every morning and evening) for new hatchlings. Hatchling lizards were individually identified, sexed by eversion of the hemipenes, measured (SVL to 0.01 mm, body mass to 0.01 g) and randomly allocated to seven terraria (600 × 450 × 340 mm, 20 mm sand substrate). Each terrarium contained two hides spaced 550 mm apart (inverted 90 mm diameter flower-pot trays, with an entrance 50 mm wide). The entrance of one hide was covered with a transparent film to prevent hatchlings’ access to one hide, but not the other (Figure 2). Because familiar environments can facilitate spatial cognition of reptiles [8], we set up the hides in the same positions in all seven terraria throughout the experiment [7]. Hatchlings (6-7/terrarium) were maintained under normoxic conditions (photoperiod 14L: 10D at 30 ± 1°C) with food and water available *ad libitum* (Figure 2).

We quantified locomotor performance (sprint speed) of hatchling lizards roughly 30 days after hatching (lizard range in age: 29-32 days) by racing each hatchling down a 100cm racetrack (marked at 20cm intervals), encouraged with a soft paintbrush. Individuals were raced three times each at 30 ± 1°C, with 1h of rest between each consecutive race. For each individual we averaged the fastest 20cm from each of the three races to quantify locomotor performance.

We then tested the cognitive abilities of hatchling lizards in the terrarium where they were fed [8,33] (Figure 2), using established methods [7]. Each lizard was tested individually four times daily for four consecutive days (n = 16 tests/lizard). For each test, we removed food, water and all individuals from the cage, and then placed an individual lizard beneath an opaque container located halfway between the two potential hides in its home cage. After 30 seconds, we lifted the container and stimulated the hatchling to run by tapping its tail with a paintbrush. A lizard successfully escaped if it fled into the open retreat within 30 seconds; any attempts to enter the closed shelter were scored as errors. If an individual was not successful within 120 seconds, it was placed inside the open retreat for 60 seconds and the test terminated. We mixed the sand substrate between each test, and lizards were maintained in their communal cage between tests. We used learning ability to assess cognition of hatchling lizards, defined as an increase in the probability of successfully escaping over tests.

We compared differences in hatching success and hatchling survival (to 30 d) among incubation regimes using chi-square tests. We used one-way ANOVAs to compare body size (SVL; at both hatching and at 30 d, when testing occurred) among oxygen treatments. To compare differences in initial egg mass, body mass at hatching and body mass at 30d among oxygen treatments; we used one-way ANCOVAs with SVL of females, initial egg mass and hatchling body mass as the covariates, respectively. To assess learning ability, we employed Generalized Estimating Equations (GEE) to test whether oxygen treatment and sex predicted the escape outcome (1 = successfully escaped; 0 = failed to escape) over 16 consecutive tests, with SVL and locomotor speed as covariates. We also conducted GEE to test whether oxygen treatment and sex determined the number of errors lizards made across trials, with SVL and locomotor speed as covariates. We selected the hyperoxic treatment group as the focal subject for analysis. We selected the model with the best fit, according to quasi-likelihood under the independence model criterion (QIC). The model with the lowest QICC value (corrected QIC value) was considered to provide the best explanation of variance in the dependent variable. Finally, we used a Logistic Regression to assess whether escape probabilities and the number of errors made by individual lizards were dependent upon the test processes. Prior to analysis of variance, we checked the raw data for normality and homogeneity of variances using Kolmogorov–Smirnov and Levene’s tests. All variables met assumptions of normality (all *p* > 0.109) and homogeneity of variance (all *p* > 0.193). Means are ± S.E. and statistical significance set at α = 0.05.

## Competing interests

The authors declared that they have no competing interests.

## Authors’ contributions

WGD and BJS designed the study. BJS, TTW and LL participated in the experiments. WGD and BJS performed statistical analyses. WGD, DAP and BJS drafted the manuscript. All authors have read and approved the final manuscript.
